# Blood-Brain Barrier Opening by Individualized Closed-Loop Feedback Control of Focused Ultrasound

**DOI:** 10.34133/2022/9867230

**Published:** 2022-04-05

**Authors:** Chih-Yen Chien, Yaoheng Yang, Yan Gong, Yimei Yue, Hong Chen

**Affiliations:** ^1^Department of Biomedical Engineering, Washington University in St. Louis, Saint Louis, Missouri 63130, USA; ^2^Department of Radiation Oncology, Washington University School of Medicine, Saint Louis, Missouri 63108, USA

## Abstract

*Objective and Impact Statement*. To develop an approach for individualized closed-loop feedback control of microbubble cavitation to achieve safe and effective focused ultrasound in combination with microbubble-induced blood-brain barrier opening (FUS-BBBO). *Introduction*. FUS-BBBO is a promising strategy for noninvasive and localized brain drug delivery with a growing number of clinical studies currently ongoing. Real-time cavitation monitoring and feedback control are critical to achieving safe and effective FUS-BBBO. However, feedback control algorithms used in the past were either open-loop or without consideration of baseline cavitation level difference among subjects. *Methods*. This study performed feedback-controlled FUS-BBBO by defining the target cavitation level based on the baseline stable cavitation level of an individual subject with “dummy” FUS sonication. The dummy FUS sonication applied FUS with a low acoustic pressure for a short duration in the presence of microbubbles to define the baseline stable cavitation level that took into consideration of individual differences in the detected cavitation emissions. FUS-BBBO was then achieved through two sonication phases: ramping-up phase to reach the target cavitation level and maintaining phase to control the stable cavitation level at the target cavitation level. *Results*. Evaluations performed in wild-type mice demonstrated that this approach achieved effective and safe trans-BBB delivery of a model drug. The drug delivery efficiency increased as the target cavitation level increased from 0.5 dB to 2 dB without causing vascular damage. Increasing the target cavitation level to 3 dB and 4 dB increased the probability of tissue damage. *Conclusions*. Safe and effective brain drug delivery was achieved using the individualized closed-loop feedback-controlled FUS-BBBO.

## 1. Introduction

The blood-brain barrier (BBB) is a natural barrier in the brain that prevents most systemically administrated therapeutic agents from reaching the brain parenchyma. Focused ultrasound (FUS) in combination with intravenously injected microbubbles for blood-brain barrier opening (FUS-BBBO) has been established as a promising technique for delivering therapeutic agents to a targeted brain region without invasive surgery. Its safety and efficacy have been demonstrated in small animals [1–6], large animals [7–10], and humans [11–18]. Cavitation is the fundamental physical mechanism of FUS-BBBO. Depending on the acoustic pressure, microbubble cavitation can range from stable cavitation to inertial cavitation. Microbubbles undergo sustained, low-amplitude volumetric oscillation (i.e., stable cavitation) at low acoustic pressures, which could increase the BBB permeability without causing any vascular damage [19, 20]. Microbubbles expand to large sizes and collapse violently (i.e., inertial cavitation) at high acoustic pressures, which could further increase the BBB permeability but may induce vascular disruption [21–24]. In order to maintain FUS exposure within a safe and effective window, passive cavitation detection (PCD)- based feedback control algorithms have been proposed for real-time monitoring of cavitation and providing feedback control of the FUS sonication pressure [12, 25–31].

The use of a feedback control algorithm to achieve safe FUS-BBBO was first demonstrated by O’Reilly and Hynynen [29]. They increased the sonication pressure until ultraharmonic signals from microbubble emissions were detected (ramping-up phase) and then decreased the acoustic pressure by 50% and maintained it at that level for the subsequent treatment in an open-loop fashion (maintaining phase). This approach considered the individual differences in the detected cavitation signals because the threshold was defined based on calibration performed for an individual subject during the ramping-up phase. The individual differences in the detected cavitation signals could arise from several factors, including variations in the in situ acoustic pressure in the brain due to differences in skull thickness and the incident angle of the FUS beam, variations in microbubble concentration and size distribution for each injection, and variations in spatial distribution of the microbubbles in the targeted brain region due to differences in vascular density, vessel size, and blood flow. This feedback control approach has been adopted by the clinical FUS device (Exablate Neuro, InSightec, Israel) and used in most of the reported FUS-BBBO clinical studies with the modification that the threshold was defined by the detection of subharmonic signals [11, 12, 15, 17], reflecting microbubble activity near the inertial cavitation threshold [28]. There are two potential limitations of this approach: the pressure ramping-up phase requires the pressure overshoot to reach the threshold, which may carry the risk of causing tissue damage; the maintaining phase uses an open-loop approach, which maintains the acoustic pressure at a fixed value throughout the maintaining phase.

Recently, several closed-loop feedback control algorithms were proposed for FUS-BBBO. For example, Sun et al. [25] developed a closed-loop algorithm using an adaptive proportional-integral controller for drug delivery across the BBB in a rat glioma model. The controller monitored the cavitation emissions throughout the experiment and adjusted the ultrasound pressure level based on the previous state of the controller and a targeted cavitation level. They defined the target cavitation level as the maximum harmonic emission level achieved without broadband detection based on prior experiments and then used the same target cavitation level for all subjects. Bing et al. [26] regulated the sonication pressure for each pulse to maintain the cavitation level within a predefined range. These studies demonstrated the capability of the proposed strategies in controlling cavitation activity in real-time in a closed-loop fashion; however, these algorithms applied the same predefined target cavitation level to all subjects without considering individual differences in the baseline cavitation signals. McDannold et al. modulated the acoustic power level until the mean harmonic signal reached a target between 6 and 7.5 dB above the noise level detected before microbubble injection and then fixed to the average pressure level that resulted in this target range for the remaining sonication [28]. Similarly, Kamimura et al. [27] developed a feedback control method using relative spectrum defined as the ratio of the instantaneous signal power spectrum after microbubble injection and the corresponding baseline power spectrum before microbubble injection. These algorithms defined the baseline cavitation levels without microbubble injection. These baseline cavitation levels did not take into consideration of individual differences caused by microbubbles. Patel et al. [32] implemented a closed-loop nonlinear state controller to control the acoustic exposure level based on passive cavitation imaging. Passive cavitation imaging enables spatially specific measurement of cavitation activity for spatial-selective feedback control of FUS-BBBO. However, it requires the use of a customized ultrasound imaging system coupled with an advanced beamforming technique for fast 2D passive cavitation imaging, which limits its broad application in FUS-BBBO.

In this study, we proposed a closed-loop feedback control algorithm for FUS-BBBO with an individualized target cavitation level defined based on the cavitation level of each subject with “dummy” FUS sonication after the injection of microbubbles. The dummy sonication applied a low acoustic pressure for a short duration at the targeted brain location after intraveneous injection of microbubbles to acquire the baseline stable cavitation signal. The target cavitation level was defined relative to the baseline stable cavitation level to control the FUS-BBBO drug delivery outcomes. The performance of the proposed approach was evaluated using wild-type mice for the delivery of a commonly used model drug, Evans blue.

## 2. Results

### 2.1. Evaluation of the Feedback Control Algorithm

**Figure 1:**
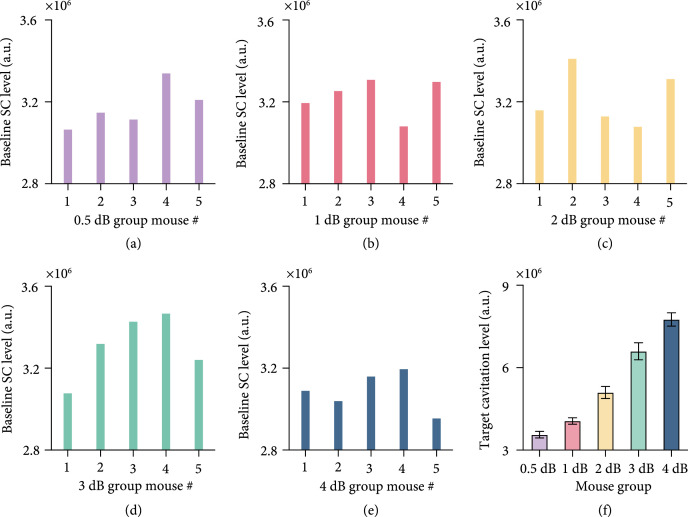
Baseline stable cavitation levels. (a–e) Baseline stable cavitation level of each subject in the five groups with different target cavitation level (i.e., 0.5 dB, 1 dB, 2 dB, 3 dB, or 4 dB). (f) The bar plot shows the mean and standard deviation of the target cavitation level for the five groups of mice.

**Figure 2:**
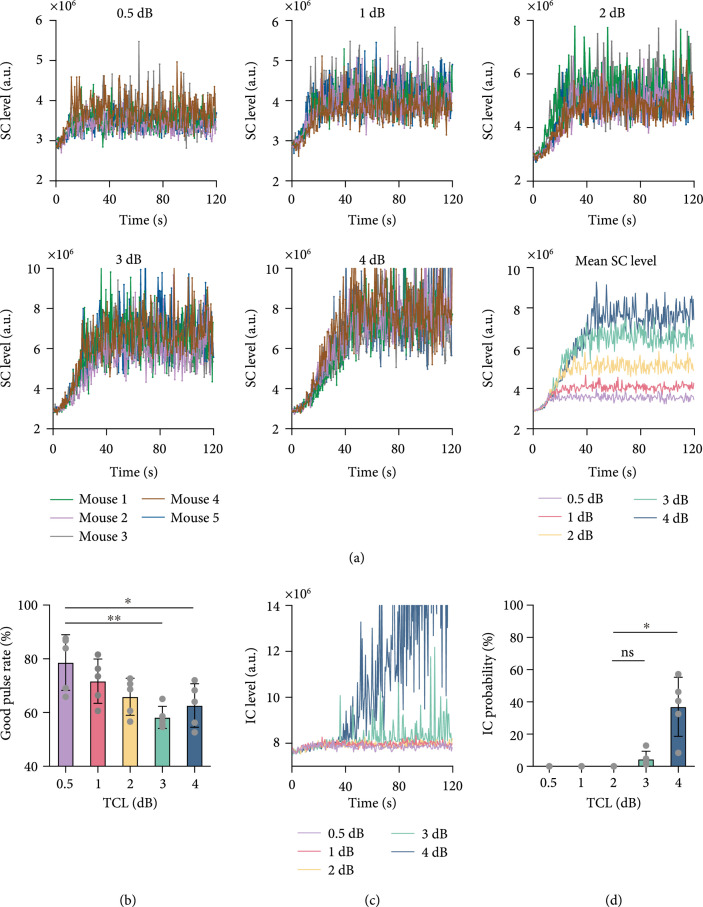
Performance of the proposed feedback control algorithm. (a) Stable cavitation level as a function of time at different target cavitation levels (i.e., 0.5 dB, 1 dB, 2 dB, 3 dB, or 4 dB above baseline stable cavitation level). Each color represents the stable cavitation level obtained from each mouse. The last graph shows the average stable cavitation level for each target cavitation level group. (b) Good pulse rate of the feedback control algorithm at each target cavitation level. (c) The average inertial cavitation level at each target cavitation level. (d) Inertial cavitation probability at different target cavitation levels. The bar plot in (b) and (d) shows the mean and standard deviation. Each circular point represents the result obtained from one mouse (Tukey’s test ${}^{*}P<0.05$; ${}^{**}P<0.01$; ns/no label: not significant).

Figures 1(a)–1(e) show the baseline stable cavitation level measured with dummy sonication in mice assigned to five groups with different target cavitation levels (0.5 dB, 1 dB, 2 dB, 3dB, and 4dB). As expected, variations in the baseline stable cavitation level were observed among different subjects. The proposed feedback control algorithm maintained the stable cavitation level at the target cavitation level that was 0.5 dB, 1 dB, 2 dB, 3 dB, or 4 dB above the baseline stable cavitation level for the individual mouse; therefore, the target stable cavitation level was different for each mouse (Figure 1(f)). The measured stable cavitation level for each mouse in each group throughout the FUS-BBBO procedure is displayed in Figure 2(a). The plot of the mean stable cavitation levels for all groups in Figure 2(a) clearly shows that the feedback control algorithm was capable of controlling the FUS sonication to maintain the stable cavitation at different levels. The stability of the control algorithm (Figure 2(b)) was 78.6%, 71.7%, 65.9%, 58.2%, and 62.6% in average for the target cavitation level of 0.5 dB, 1 dB, 2 dB, 3 dB, and 4 dB, respectively. The inertial cavitation level was monitored in all studies, and Figure 2(c) presents the measured mean inertial cavitation levels for each group. Average inertial cavitation probability was 0%, 0%, 0%, 4.5%, and 37.0% for the target cavitation level of 0.5 dB, 1 dB, 2 dB, 3 dB, and 4 dB, respectively (Figure 2(d)). Inertial cavitation probability was significantly higher at 4 dB than other groups.

### 2.2. Quantification of the Evans Blue Delivery Outcome

**Figure 3:**
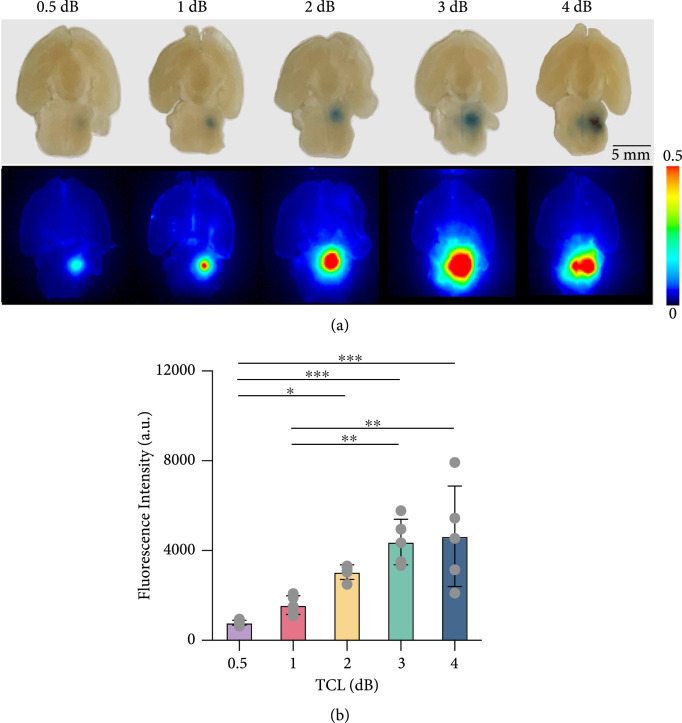
Evans blue delivery outcomes. (a) Representative photographs and corresponding fluorescence images of brain slices at target cavitation level of 0.5 dB, 1 dB, 2 dB, 3 dB, and 4 dB, respectively. (b) The fluorescence intensity at each target cavitation level. The bar plot in (b) shows the mean and standard deviation. Each circular point represents the result obtained from each mouse (Tukey’s test ${}^{*}P<0.05$; ${}^{**}P<0.01$; ${}^{***}P<0.001$).

Figure 3(a) shows photographs of representative brain slices and corresponding fluorescence images at target cavitation level of 0.5 dB, 1 dB, 2 dB, 3 dB, and 4 dB, respectively. Compared with the 0.5 dB group, the delivered Evans blue fluorescence intensity increased in an average of 2.0-fold, 3.9-fold, 5.6-fold, and 5.9-fold at 1 dB, 2 dB, 3 dB, and 4 dB, respectively (Figure 3(b)). These data showed that the average FUS-BBBO delivery efficiency was monotonically increased when the target cavitation level was increased from 0.5 dB to 3 dB, but this trend was not maintained when target cavitation level was increased to 4 dB.

### 2.3. Safety Evaluation

**Figure 4:**
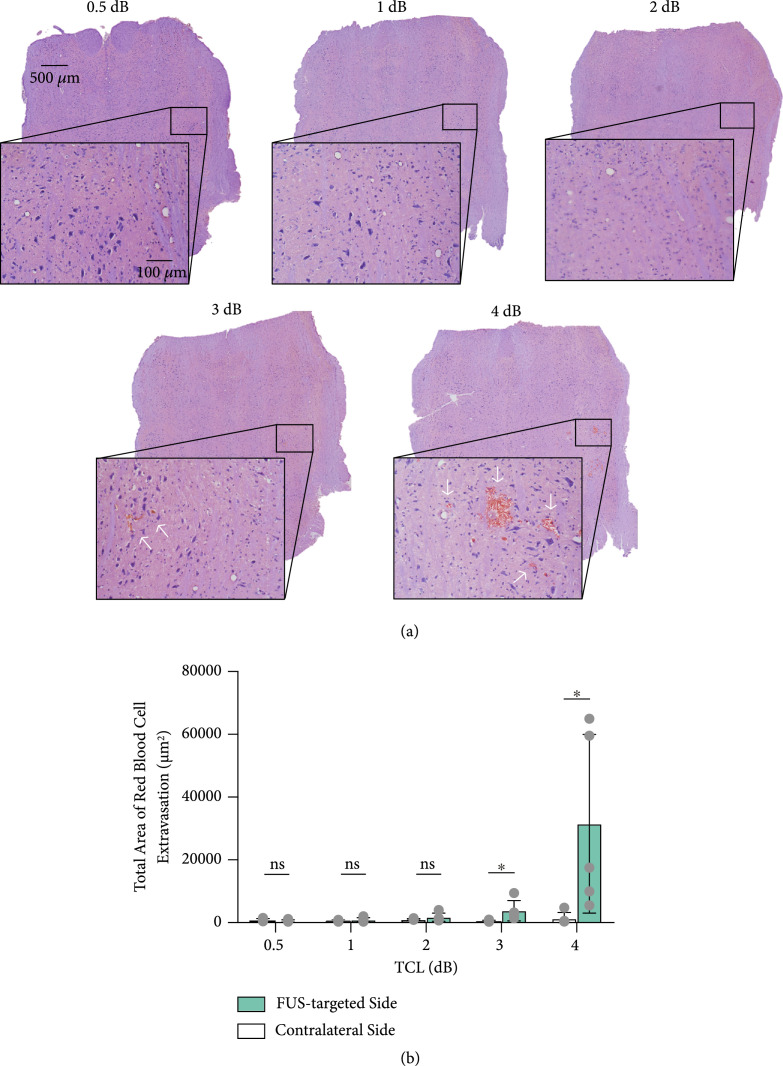
Safety analysis. (a) Representative H&E staining of the FUS-targeted side of the brainstem at each target cavitation level. (b) Comparison of hemorrhage area of each target cavitation level and the contralateral side. The bar plot in (b) shows the mean and standard deviation. Each circular point represents the result obtained from each mouse (Tukey’s test ${}^{*}P<0.05$; ns: not significant).

Figure 4(a) shows the representative hematoxylin and eosin (H&E) staining of the brainstem for each group. FUS was targeted at the right side of the brainstem, and the contralateral side was used as the control. As shown by the lower and higher magnification images, no hemorrhage was observed in the 0.5 dB, 1 dB, and 2 dB cases. Mild tissue damage was found in the 3 dB case, and relatively severe tissue damage was found in the 4 dB case. Group analysis found no significant difference between the FUS-targeted side and contralateral nontreated side in the 0.5 dB, 1 dB, and 2 dB groups (Figure 4(b)). Hemorrhage was observed within the FUS-targeted region in 2 out of 5 mice in the 3 dB group and 5 out of 5 mice in the 4 dB group (3 dB: FUS-targeted side versus contralateral side, P=0.0496; 4 dB: FUS-targeted side versus contralateral side, P=0.0455).

## 3. Discussion

This study achieved reliable and safe FUS-BBBO using an individualized closed-loop feedback control strategy. FUS-BBBO depends on interactions among three key factors: ultrasound, microbubbles, and cerebral vasculature [33]. Our proposed approach defined the target cavitation level based on FUS sonication at a low pressure (0.2 MPa as measured in water, and the in situ acoustic pressure was estimated to be 0.16 MPa considering the mouse skull attenuation was about 18% [34]) for a few seconds (5 s). This dummy sonication is below the exposure energy needed to induce BBB opening [35]. At the same time, it allows the feedback control algorithm to take into consideration of individual differences because the acoustic emissions detected with the dummy sonication were affected by all these three key factors: (1) ultrasound: the individual differences in the skull thickness and incident angle of the FUS beam on the skull affect the in situ acoustic pressure; (2) microbubbles: variations in the preparation and infusion procedures affect the injected microbubble concentration and size distribution; and (3) cerebral vasculature: differences in vascular density, vessel size, and blood flow affect the amount of microbubbles that reach the FUS-targeted brain region. Our experimental data confirmed that variations in the baseline stable cavitation level were detected among different mice (Figures 1(a)–1(e)). Due to differences in the baseline stable cavitation level of each subject, the targeted stable cavitation level of each subject was different under the same target cavitation level (Figure 1(f)).

The approach proposed by O’Reilly and Hynynen [29] and later adopted in the clinical trials also considered the individual differences in the detected cavitation signals as the threshold was defined based on calibration performed with FUS sonication in the presence of microbubbles for an individual subject. However, the approach used in the clinical system required pressure overshoot until detecting microbubble activity near inertial cavitation threshold in the pressure ramping-up phase. The pressure overshoot increases the risk of tissue damage. Others have proposed an alternative strategy to avoid overexposure in the ramping-up phase by defined the target cavitation level relative to the baseline stable cavitation level detected before microbubble injection [27, 28]. This strategy could separate microbubble emission from signals generated by other sources (e.g., bubbles trapped in the water/gel coupling medium), but it did not take into consideration the variations in microbubbles and cerebral vasculature among different subjects.

Our proposed closed-loop feedback control algorithm was capable of maintaining the stable cavitation level at the target cavitation level with high stability. Although several PCD-based feedback control algorithms have been introduced, there is only one paper by Sun et al. [25] that quantified the controller performance. This paper used the good burst rate to measure the percentage of pulses with harmonic emissions within a predefined desired range using a PCD-based closed-loop feedback controller. They found that the good burst rate achieved with microbubble infusion was on average about 45% at a pulse repetition frequency of 1 Hz and about 70% at a pulse repetition frequency of 4 Hz. We quantified the stability of our proposed approach using the good pulse rate and found it was within 58.2–78.6% for target cavitation levels within the range of 0.5 dB to 4 dB. Based on this measurement, the performance of our closed-loop feedback controller was comparable to that proposed by Sun et al. [25].

Our data suggest that the selection of the optimal target cavitation level needs to consider the stability of the feedback controller and the FUS-BBBO delivery outcome and safety. Although no significant difference was detected among the good pulse rates for the five groups, increasing target cavitation level was observed to be associated with a decreasing trend in the good pulse rate (Figure 2(b)), indicating a decrease in the controllability. Increasing the target cavitation level within the range of 0.5 dB to 3 dB induced an approximately linear increase in the Evans blue fluorescence intensity. No tissue damage was observed at target cavitation level of 0.5 dB, 1 dB, and 2 dB, and hemorrhage was observed in 2/5 mice at target cavitation level of 3 dB. Further increasing target cavitation level to 4 dB was associated with the presence of inertial cavitation (Figure 2(d)) and vascular damage in all five mice (Figure 4(b)). The consistent observation of vascular damage at 4 dB may explain the observation that the Evans blue delivery efficiency did not further increase as the target cavitation level was increased to 4 dB. By integrating these factors, a target cavitation level of 2 dB was considered to be the optimal level for efficient and safe FUS-BBBO delivery of Evans blue to mouse brainstem based on findings from this study. It needs to point out that 2 dB cannot be considered as the optimal target cavitation level for all conditions, but the method we used to define the optimal target cavitation level can be extended to FUS-BBBO in other brain locations and even other species. For example, the optimal target cavitation level for FUS-BBBO in human can be defined by the target cavitation level that achieves the maximum BBB disruption as measured by contrast-enhanced MRI without inducing detectable inertial cavitation. Future studies are needed to evaluate the performance of our proposed approach in controlling FUS-BBBO in other conditions, for example, different brain locations, different therapeutic agents, and different species (e.g., larger animals and humans). Once fully validated, our proposed approach can be adopted by clinical FUS devices to control the FUS-BBBO studies in patients. Future studies could also combine the feedback control of stable cavitation level with the control of sonication duration to modulate the cumulative cavitation dose [36]. It is also worth pointing out that inertial cavitation was monitored in this study but not included in the proposed feedback control algorithm. Future studies could integrate inertial cavitation in the feedback control algorithm to decrease the sonication pressure or stop the treatment when inertial cavitation is detected to avoid tissue damage [27].

This study used stable cavitation level to control FUS-BBBO. In addition to stable cavitation level, inertial cavitation probability could be also used for the control of FUS-BBBO; however, it has two limitations. First, it relies on the detection of inertial cavitation. The presence of inertial cavitation can be associated with tissue damage. Therefore, the use of inertial cavitation probability for feedback control increases risk of tissue damage. Secondly, different from stable cavitation-based feedback control that can tune the stable cavitation level to achieve different levels of drug delivery, inertial cavitation probability lacks the capability to tune the drug delivery level.

In conclusion, this study achieved reliable and safe FUS-BBBO using an individualized closed-loop feedback control algorithm at selected target cavitation levels. The use of FUS sonication at a low pressure and short duration to establish the target cavitation level provided a strategy that took into consideration of individual differences in the detected cavitation signals and avoided overexposure. The proposed feedback control algorithm had high stability and successfully controlled the FUS-BBBO drug delivery outcomes. The optimal target cavitation level was selected by considering the performance of the controller and the FUS-BBBO delivery efficiency and safety. Findings from this study highlight the importance of controlling the FUS exposure to achieve efficient and safe BBBO.

## 4. Materials and Methods

### 4.1. Experimental Design

**Figure 5:**
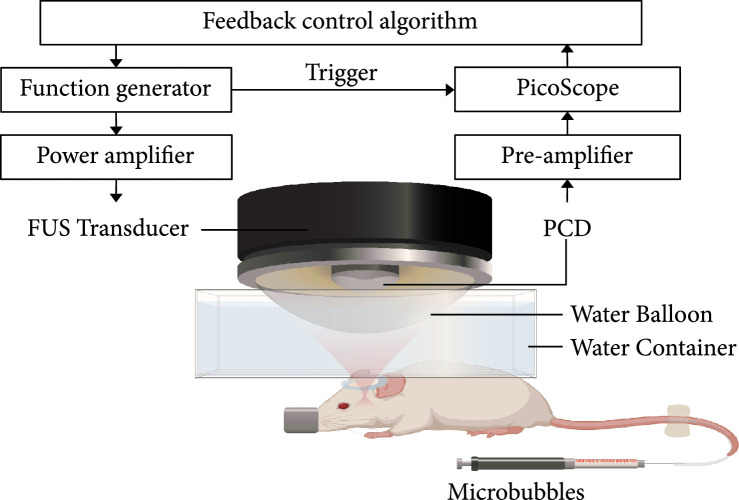
Illustration of the feedback-controlled FUS system. The experiment setup was composed of three parts: (1) transmission: FUS transducer, function generator, and power amplifier; (2) receiving: PCD, preamplifier, and PicoScope; and (3) feedback control: a customized MATLAB program for the feedback control.

A single-element FUS transducer with an aperture of 75 mm and a radius of curvature of 60 mm and a center opening of 25 mm in diameter were used in this study (Figure 5). The FUS transducer was impedance matched to operate at 1.5 MHz and driven by an arbitrary waveform generator (Agilent 33500B; Agilent Technologies, Loveland, CO, USA) that was connected to a 53 dB power amplifier (1020 L; E&I, Rochester, NY, USA). The FUS transducer was attached to a 3D stage. The acoustic pressure fields generated by the FUS transducer were calibrated using a needle hydrophone (HNP-0200; Onda Inc., Sunnyvale, USA) in a degassed water tank. The axial and lateral full-width-at-half-maximum (FWHM) dimensions of the FUS transducer were 8.3 mm and 1.1 mm, respectively. The peak negative pressures of the FUS transducer at different voltage input levels were measured at the focus of the transducer in a water tank. A 3D-printed bar with a sharp tip was manufactured so that when the FUS transducer was switched to the bar, the tip of the bar almost touched the tip of the hydrophone. The pointer was then used to indicate the FUS focus to facilitate precise targeting of a specific brain location. The tip of the pointer was moved by the 3D stage to be aligned with the lambda on the mouse skull, which was visible through the mouse skin. The pointer was then switched to the FUS transducer. The 3D-printed holder and transducer housing and pointer were manufactured to fit tightly with each other. A surface leveler was also used every time after switching the pointer to the transducer to confirm that the transducer was placed in the horizontal plane without tilting. The transducer was moved 1 mm lateral and 1 mm posterior and 4 mm ventral to target the brainstem, which was selected as the targeted brain location. A single-element ultrasound transducer (I5P10, Guangzhou, China) with a center frequency of 4.7 MHz and a bandwidth (-6 dB) of 78.6% was inserted through the center hole of the FUS transducer and confocally aligned with it using a 3D-printed housing. This transducer was used as a PCD to acquire cavitation emissions from the microbubbles during FUS sonication. It was connected to a 22 dB preamplifier and then a PicoScope (5244B, Pico Technology, Cambridgeshire, UK). The PicoScope was triggered by the arbitrary waveform generator to synchronize PCD data acquisition with the FUS sonication. The signal acquired by the PCD was sampled at 40 MHz. All the equipment was controlled by a personal computer using a custom MATLAB program. The MATLAB code for the feedback control is uploaded to GitHub and permitted free access at (https://github.com/ChenUltrasoundLabWUSTL/Public-Feedback-Control.git).

### 4.2. FUS-BBBO under Real-Time Closed-Loop Feedback Control

**Figure 6:**
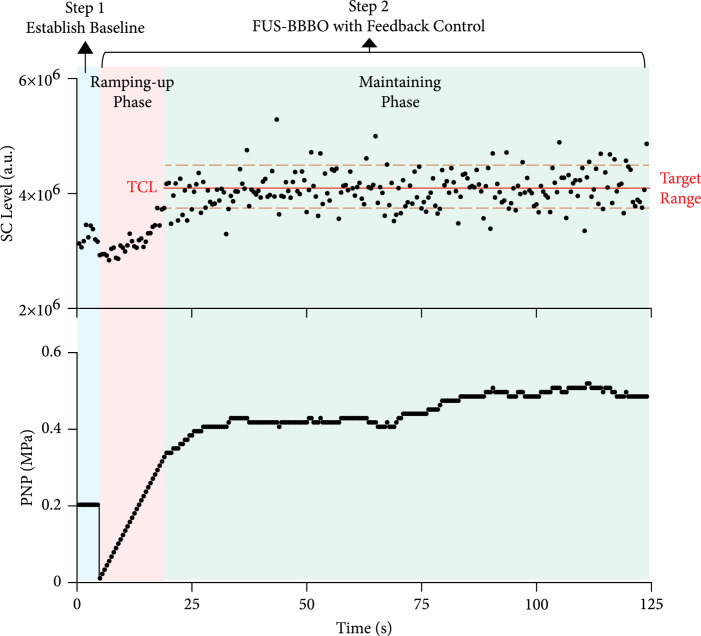
Illustration of the feedback control algorithm. Microbubble infusion was started 15 seconds before FUS sonication and lasted until the end of FUS sonication. During FUS sonication with microbubble infusion, cavitation was monitored by PCD in real-time. The baseline stable cavitation level for each mouse was defined using 10 repeated PCD measurements acquired during dummy FUS sonication. Once target cavitation level was defined relative to baseline stable cavitation level, FUS sonication was performed with the feedback control algorithm in a two-phase process: the pressure ramping-up phase to reach target cavitation level and maintaining phase to keep stable cavitation level within the target range.

The microbubble contrast agent (Definity, Lantheus Medical Imaging, North Billerica, MA) was diluted using sterile saline to a final concentration of approximately $8\times 10^{8}$ number of microbubbles per mL. This concentration was close to the clinically approved number concentration of Definity. The manufacturer recommendation infusion dose for activated Definity is to add 1.3 mL Definity to 50 mL of saline. Each 1 mL of activated Definity solution contains a maximum of $1.2\times 10^{10}$ microbubbles according to the datasheet provided by the manufacter. Therefore, the concentration of the diluted Definity is estimated to be $3.12\times 10^{8}$ microbubbles per mL according to information provided by the manufacturer. The concentration ($8\times 10^{8}$ microbubbles per mL) used in our study was at the same order of magnitude as the recommended dose. This number concentration was used in previous FUS-BBBO studies in mice [5, 22]. The diluted microbubbles (volume=30 *μ*L) were injected intravenously through the tail vein catheter. The injection was performed using a computer-controlled syringe pump (NE-1600; New Era Pump Systems Inc.). Microbubble infusion was started 15 s before FUS sonication to allow microbubbles to flow through the tail vein catheter and reach the mouse brain. The infusion lasted until the end of sonication at a constant rate of 12.8 *μ*L/min. All mice were treated by FUS with output pressure controlled in real-time using the proposed PCD-based closed-loop feedback control algorithm. The treatment procedure follows a two-step process, as illustrated in Figure 6.

#### 4.2.1. Step 1: Establish Baseline Stable Cavitation Level

The baseline stable cavitation level for each mouse was defined based on the dummy FUS sonication with microbubble injection. The dummy FUS sonication was performed using a pulse repetition frequency of 2 Hz, a pulse length of 6.7 ms (i.e., duty cycle: 1.3%), and a sonication duration of 5 s. The output pressure of FUS was 0.2 MPa (all pressures reported in this study were the peak negative pressures calibrated in water). This pressure was selected because it was the lowest pressure at which the microbubble cavitation signal was higher than the cavitation level measured with FUS sonication without microbubble infusion. During the sonication by each FUS pulse, acoustic emission from microbubbles was recorded by the PCD and processed by the fast Fourier transform (FFT) algorithm. The stable cavitation level was calculated by summing the magnitude of spectrum within a ±20 kHz bandwidth centered at the third harmonic (i.e., 4.5 MHz) of the FUS transducer. The harmonic emission was chosen because it represents the stable cavitation activities of microbubbles, and the third harmonic was selected because among all the harmonic frequencies, it was closest to the center frequency of the PCD transducer. Ten PCD signals were acquired, and the average stable cavitation level calculated from these ten signals was used to define the baseline stable cavitation level.

#### 4.2.2. Step 2: FUS-BBBO with Real-Time Feedback Control

After establishing the baseline stable cavitation level, FUS-BBBO was performed with real-time feedback control. During FUS sonication with microbubble infusion, cavitation was monitored by PCD in real-time, and a custom closed-loop feedback control algorithm was used to control the stable cavitation level to be at different target cavitation levels defined to be 0.5 dB, 1 dB, 2 dB, 3 dB, or 4 dB above the baseline stable cavitation level. Different levels of target cavitation levels were selected to investigate the dependencies of FUS-BBBO drug delivery outcome and safety on the target cavitation level. The proposed feedback control algorithm consisted of two sonication phases: the ramping-up phase and maintaining phase (Figure 6). The ramping-up phase started from 0 MPa and increased pulse by pulse with a step of 13 kPa until the stable cavitation level reached the target cavitation level. Then, the control algorithm switched to the maintaining phase with the acoustic pressure adjusted to maintain stable cavitation level within the target range (i.e., target cavitation level±tolerance range) until the end of the sonication. The tolerance range was set to ±0.4 dB to reduce the sensitivity to noise. If the stable cavitation level was located within the range of target cavitation level±tolerance range, the FUS output pressure was kept the same. For the case that stable cavitation level was higher or lower than target cavitation level±tolerance range, FUS output pressure of the next pulse was decreased or increased by the step size (13 kPa) immediately. The step size (13 kPa) was the minimum step size of the arbitrary waveform generator and was used to achieve fine adjustment.

### 4.3. Feedback Controller Characterization

The stability of the feedback control algorithm was determined by the good pulse rate, which measured the percentage of FUS pulses during which the stable cavitation level was within the target range in the maintaining phase. Higher good pulse rate represents higher controllability of the cavitation activities. Inertial cavitation level was also quantified based on the acquired cavitation signals to serve as a safety check. Inertial cavitation level was calculated by summing the magnitude of spectrum within 3.3±0.02 MHz. These frequencies were chosen to quantify the level of the broadband signals by avoiding harmonics and ultraharmonics. The presence of an inertial cavitation event was defined when the inertial cavitation level was over 1 dB above the baseline inertial cavitation level quantified based on the signals acquired during dummy FUS sonication. Inertial cavitation probability was calculated by the percentage of inertial cavitation events that were present during the maintaining phase. Higher inertial cavitation probability indicates higher occurring of inertial cavitation events and higher potential of tissue damage [22].

### 4.4. Drug Delivery Outcome Evaluation

Evans blue, a widely used agent to evaluate BBB permeability changes, was used as a model drug in this study. Mice were intravenously injected with 30 *μ*L of 4% Evans blue immediately after FUS sonication. Mice were sacrificed and perfused 30 minutes after sonication. Mouse brains were then harvested and fixed using 4% paraformaldehyde. The extracted whole brains were sectioned into 1 mm thick slices in the horizontal plane and examined by the Licor Pearl small animal imaging system (LI-COR Biosciences, Lincoln, NE) for imaging Evans blue. The exposure time for fluorescence imaging was kept the same for imaging all the brain slices. For each mouse brain, a region of interest (ROI) was selected to cover the whole brainstem and quantified using the LI-COR Image Studio Lite software to calculate the sum of the fluorescence intensity within the ROI. The fluorescence intensity was used to represent the Evans blue delivery concentration at the target region, indicating FUS-BBBO drug delivery efficiency. The delivered Evans blue increasement was calculated by dividing the fluorescence intensity of the 1 dB, 2 dB, 3 dB, and 4 dB group individually by that of the 0.5 dB group.

### 4.5. Safety Evaluation by Histologic Analysis

Histologic examination was performed on all mice using hematoxylin and eosin (H&E) staining. Specifically, after fluorescence imaging, the brain slices containing the targeted brainstem were fixed in 4% paraformaldehyde overnight, followed by 30% sucrose as cryoprotectant and cryostat embedding at -20°C. The brain slices were sectioned horizontally into 10 *μ*m sections and stained with H&E. Digital images of tissue sections were obtained using an all-in-one microscope (BZ-X810, Keyence, Osaka, Japan). The hemorrhage area was extracted based on pixel hue by the built-in software of BZ-X810. The total area of red blood cell extravasation was calculated by summing all the identified pixels in the FUS-targeted side of the brainstem. The contralateral brain area without FUS sonication was used as the control.

### 4.6. Animal Studies

All animal studies were reviewed and approved by the Institutional Animal Care and Use Committee of Washington University in St. Louis in accordance with the National Institutes of Health Guidelines for Animal Research. The animals were housed in a room maintained at 22°C and 55% relative humidity, with a 12 h/12 h light/dark cycle and access to standard laboratory chow and water. A total of 25 Swiss mice (8–10 weeks, ~25 g body weight, female, Charles River Laboratory, Wilmington, MA, USA) were randomly assigned into five groups (n=5 for each group) to evaluate five different target cavitation levels using the proposed algorithm. During all experiments, mice were anesthetized with 1.5–2% isoflurane and stabilized using a stereotaxic apparatus (Kopf, Tujunga, CA, USA). A heating pad with a temperature kept at ~38°C was used to maintain the mouse body temperature. Mice were prepared for FUS sonication by removing fur on top of the head with a depilatory cream (Nair, Church & Dwight Co., NJ, USA) and coupled to a water container using ultrasound gel. A catheter was placed into the tail vein for microbubbles and Evans blue injection.

### 4.7. Statistical Analysis

Statistical analyses were performed using GraphPad Prism (Version 9.0, La Jolla, CA, USA). Differences among multiple groups were determined using one-way ANOVA followed by the Tukey’s test for group-wise comparisons. We performed normality test before ANOVA. A one-sample t-test was performed if the samples did not pass normality test. P value < 0.05 was used to determine statistical significance. The differences in the hemorrhage area between FUS-targeted side and contralateral side were determined using an unpaired two-tailed Student’s t-test. P value < 0.05 was used to determine statistical significance.

## Data Availability

The results used to support the findings of this study are included within the article, and the code used to acquire the data of this study are available at https://github.com/ChenUltrasoundLabWUSTL/Public-Feedback-Control.git. The raw datasets generated during the study are available for research purposes from the corresponding author on reasonable request.
